# ^99^TcO_4_^−^ remediation by a cationic polymeric network

**DOI:** 10.1038/s41467-018-05380-5

**Published:** 2018-08-01

**Authors:** Jie Li, Xing Dai, Lin Zhu, Chao Xu, Duo Zhang, Mark A. Silver, Peng Li, Lanhua Chen, Yongzhong Li, Douwen Zuo, Hui Zhang, Chengliang Xiao, Jing Chen, Juan Diwu, Omar K. Farha, Thomas E. Albrecht-Schmitt, Zhifang Chai, Shuao Wang

**Affiliations:** 1 State Key Laboratory of Radiation Medicine and Protection, School for Radiological and Interdisciplinary Sciences (RAD-X) and Collaborative Innovation Center of Radiation Medicine of Jiangsu Higher Education Institutions, Soochow University, 215123 Suzhou, China; 2 Collaborative Innovation Center of Advanced Nuclear Energy Technology, Institute of Nuclear and New Energy Technology, Tsinghua University, 100084 Beijing, China; 3 Department of Chemistry, Northwestern University, 2145 Sheridan Road, Evanston, IL 60208 USA; 4 Suzhou CNNC Huadong Radiation Co., Ltd, 215222 Suzhou, China; 5 CGN Dasheng Electron Accelerator Technology Co., Ltd, 215212 Suzhou, China; 6 State Key Laboratory of Functional Materials for Informatics, Shanghai Institute of Microsystem and Information Technology, Chinese Academy of Sciences, 200050 Shanghai, China; 7 Department of Chemistry, Faculty of Science, King Abdulaziz University, Jeddah, 21589 Saudi Arabia; 8 Department of Chemistry and Biochemistry, Florida State University, 95 Chieftain Way, Tallahassee, FL 32306 USA

## Abstract

Direct removal of ^99^TcO_4_^−^ from the highly acidic solution of used nuclear fuel is highly beneficial for the recovery of uranium and plutonium and more importantly aids in the elimination of ^99^Tc discharge into the environment. However, this task represents a huge challenge given the combined extreme conditions of super acidity, high ionic strength, and strong radiation field. Here we overcome this challenge using a cationic polymeric network with significant TcO_4_^−^ uptake capabilities in four aspects: the fastest sorption kinetics, the highest sorption capacity, the most promising uptake performance from highly acidic solutions, and excellent radiation-resistance and hydrolytic stability among all anion sorbent materials reported. In addition, this material is fully recyclable for multiple sorption/desorption trials, making it extremely attractive for waste partitioning and emergency remediation. The excellent TcO_4_^−^ uptake capability is elucidated by X-ray absorption spectroscopy, solid-state NMR measurement, and density functional theory analysis on anion coordination and bonding.

## Abstract (web summary)

Direct removal of ^99^TcO_4_^− ^from highly radioactive and acidic nuclear waste solutions is beneficial for uranium and plutonium recovery and radioactive pollution control but this represents a huge challenge. Here the authors show a cationic polymeric network with high ^99^TcO_4_^−^ sorption capability and stability.

## Introduction

Uranium/plutonium fission-based nuclear energy is currently one of the most optimal choices for the partial replacement of fossil fuel energy, but faces several key hurdles for further development^1–4^. For instance, searching for efficient, reliable, and economically effective strategies for used fuel reprocessing and waste disposal are still in the stage of scientific and technological exploration. Nuclear safety is another concern because several nuclear accidents have occurred in the past several decades and the resulting contamination of tremendous amounts of radioisotopes with combined radiotoxicity and chemotoxicity in the environment is a severe issue. Among them, technetium-99 (^99^Tc) is a β-emitting radionuclide with a long half-life of 2.13 × 10^5^ years and can be produced in high fission yield (~6%) in a typical uranium fission reactor leading to a large inventory in used nuclear fuel and waste^5^. It predominately exists in the nuclear fuel cycle and in the environment as the pertechnetate anion (TcO_4_^−^), which exhibits high solubility and a nearly non-complexing nature, giving rise to its high environmental mobility with transportation velocity being almost identical as that of groundwater^5,6^. Tc(VII) complexes are volatile during nuclear waste vitrification process, producing problems for the off-gas system design^7^. Another serious issue associated with this radioisotope is its capability to greatly interface with the solvent extraction/back extraction process of uranium, neptunium, and plutonium through catalytic redox reactions, generating a notable barrier on the valence state control of these key components in the fuel cycle^8,9^. Therefore, it would be ideal that TcO_4_^−^ can be separated at the first stage once used nuclear fuel rods are dissolved in highly concentrated nitric acid solutions even before the plutonium uranium redox extraction (PUREX) process. This is further beneficial because this would eliminate ^99^Tc discharge into the environment during subsequent waste managing processes. However, this task represents a huge challenge given the combined extreme condition of super acidity (e.g., 3 M nitric acid), high ionic strength (large excess of competing anions), and strong ionizing radiation field (β, γ, and neutron irradiations, etc.).

Although there has been a tremendous amount of effort being paid to designing high-performance anion-exchange materials tailored for the application of TcO_4_^−^ uptake^10^, combined features of ultrastability in acids, decent radiation-resistance, high TcO_4_^−^ uptake kinetics/capacity, and excellent sorption selectivity that are required for the aforementioned task have never been integrated into a single material. For example, traditional commercialized polymeric anion-exchange resins exhibit efficient removal of TcO_4_^−^ even in acidic conditions^11–13^, but are not radiation-resistant. This clear drawback is greatly amplified given their anion-exchange kinetics are generally quite slow, leading to significant radiation dose received during the long sorption time and subsequent radiation-induced damage of the materials. Purely inorganic cationic materials, such as layered double hydroxides (LDHs)^14,15^, layered rare-earth hydroxides (LRHs)^16^, and thorium borates (NDTB-1)^17,18^, may possess enhanced radiation-resistance, but their relatively low sorption capacity and poor selectivity toward TcO_4_^−^ impedes real applications. Recently, cationic metal–organic frameworks (MOFs) built from transition metal cations and neutral soft ligands with exchangeable anions in the pores are promising candidates to sequester TcO_4_^−^from aqueous solution^19–22^, because they display fast sorption kinetics, high capacity, excellent selectivity, and great radiation-resistance. However, none of these materials survives in highly acidic conditions. Therefore, these materials may exhibit potentials in environmental remediation at relatively neutral pHs, but certainly not in fuel reprocessing processes.

Polymeric networks built by repeating organic building blocks have been intensively investigated recently in the area of environmental pollution remediation^23–30^. They are endowed with a series of excellent features similar with MOFs including predictable reticular synthesis and structure type, controllable pore size/shape/charge, and facile functionalization tailored for trapping targeted environmental pollutants^31–37^. More importantly, these materials exhibit superior hydrolytic stability even in highly acidic/basic solutions^38–40^, a clear advantage not possessed by the majority of MOFs. In addition, polymeric network equipped with relatively large conjugated fragments in the structure may also show enhanced radiation-resistance compared to traditional polymeric anion-exchange resins because they can effectively stabilize radiation-induced radical intermediates, another merit critical for nuclear-related applications, especially used fuel reprocessing. Here we report a cationic polymeric network (CPN), SCU-CPN-1 (SCU = Soochow University), which substantially overcomes the long-term challenge of TcO_4_^−^ separation under the extreme conditions of super acidity, strong irradiation field, and high ionic strength.

## Results

### Characterization

**Fig. 1:**
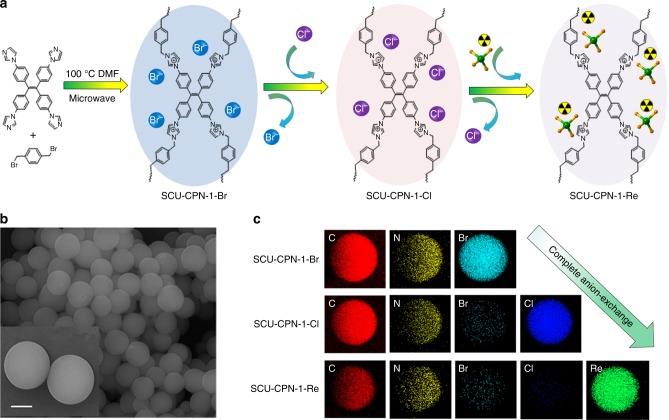
Preparation and electron microscopic characterizations of SCU-CPN-1. **a** Synthesis route of SCU-CPN-1 and its anion-exchange applications. **b** SEM image of SCU-CPN-1-Br, showing the particle size is ca. 2 μm. **c** EDS mapping of SCU-CPN-1-Br, SCU-CPN-1-Cl, and SCU-CPN-1-Re, qualitatively indicative of the complete anion-exchange process. Scale bar = 1 μm

**Fig. 2:**
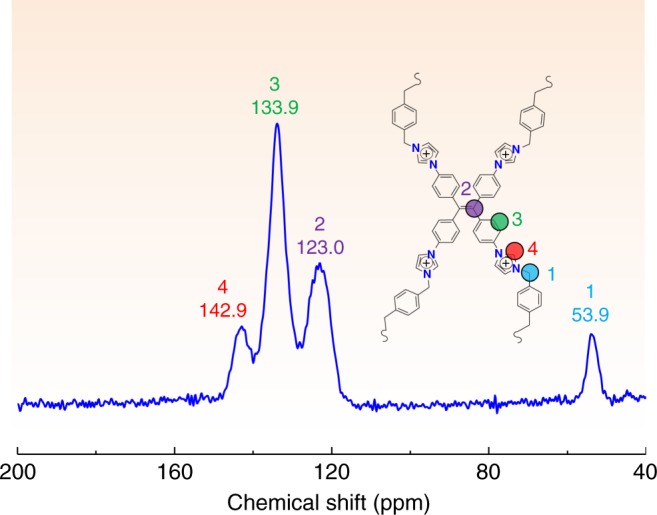
NMR analysis. Solid-state ^13^C NMR spectrum of SCU-CPN-1

SCU-CPN-1-Br was derived from the quaternization reaction between 1,1,2,2-tetrakis(4-(imidazolyl-4-yl)phenyl)ethene (TIPE) and 1,4-bis(bromomethyl)benzene (BBB) (Fig. 1a). The degree of cross-linking can be quantified by the ReO_4_^−^ sorption capacity and the Br^−^ residual after anion-exchange discussed later. SEM (Fig. 1b) images show a uniform spherical morphology with a diameter of ~2 μm. PXRD measurement confirms the amorphous nature of this material (Supplementary Fig. 1), which is similar to many other CPN materials reported^41,42^. The structure of SCU-CPN-1-Br can be best described as a cationic polymeric network consisting of TIPE and BBB moieties with exchangeable Br^−^ filled in the pores, supported from combined EDS, FT-IR, and solid-state NMR studies. In the FT-IR spectrum, the characteristic peaks of 1074 cm^−1^ correspond to the quaternary imidazolium species (Supplementary Fig. 2)^43^. The characteristic peaks at 53.9, 123, 133.9, and 142.9 ppm in the solid-state ^13^C NMR spectrum of SUC-CPN-1 (Fig. 2) can be ascribed to the *sp*^3^-hybridized carbon atoms of methylene, ethylene, *sp*^2^-hybridized carbon atoms on benzene rings, and the carbon atoms bound to the nitrogen atom, respectively. Thermogravimetric analysis shows that SCU-CPN-1-Br is stable up to 300 °C (Supplementary Fig. 3). The initial weight loss below 150 °C corresponds to the removal of water and *N*,*N*-dimethyl formamide (DMF) molecules. The N_2_ sorption isotherm measured at 77 K does not yield any noticeable uptake amount, initially suggesting a non-porous structure of SCU-CPN-1, likely because the charge-balancing anions effectively block the transportation pathway of the gas molecule. However, this is not the case for the anion-exchange process demonstrated below.

### Sorption kinetics analysis

Before anion-exchange studies, SCU-CPN-1-Br was immersed into saturated NaCl solutions to replace toxic Br^−^ with Cl^−^ (SCU-CPN-1). The anion-exchange process was confirmed by TEM-EDS mapping (Fig. 1c) and SEM-EDS (Supplementary Fig. 4) analysis. The Br^−^ residual after anion-exchange is due to the incomplete quaternization reaction in the material synthesis, because Cl^−^ can be fully exchanged with ReO_4_^−^ (see discussion below). This residue amount can be therefore used for calculation of degree of cross-linking to be 96.9 ± 0.2%. SCU-CPN-1 also facilitates the complete exchange with NO_3_^−^ (SCU-CPN-1-NO_3_) (Supplementary Fig. 5), which is beneficial on used fuel repossessing applications because Cl^−^ is often not desired in these processes given its redox property toward several actinides. It should be noted that the anion-exchange properties of SCU-CPN-1-Cl and SCU-CPN-1-NO_3_ samples are almost identical.

**Fig. 3:**
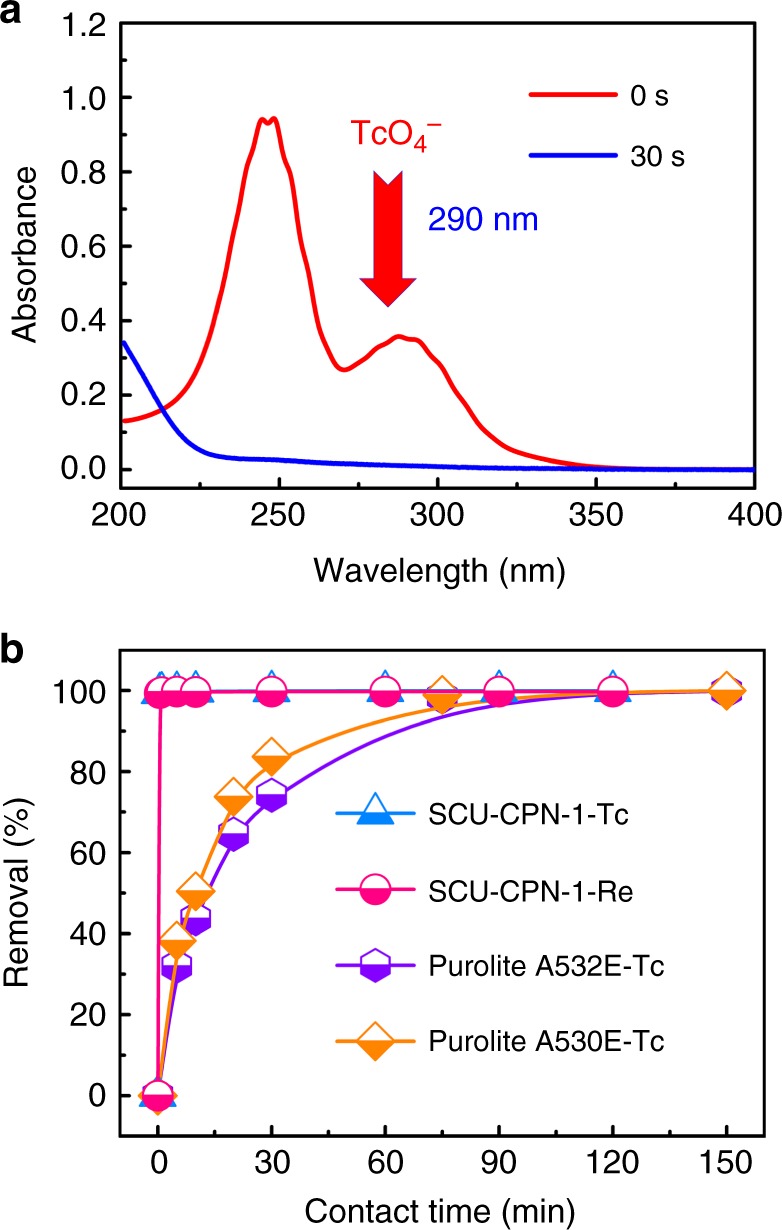
Sorption kinetics study of SCU-CPN-1. **a** UV–vis spectra of TcO_4_^−^ solution during the anion-exchange with SCU-CPN-1. **b** Sorption kinetics of TcO_4_^−^ by SCU-CPN-1 compared with Purolite A530E and Purolite A532E

TcO_4_^−^ uptake experiments were initially performed by contacting 10 mg samples of SCU-CPN-1 with a 10 ml aqueous solution containing 28 ppm ^99^TcO_4_^−^. The concentration of TcO_4_^−^in aqueous solution was monitored by its characteristic absorption feature at 290 nm in the UV–vis spectra (Fig. 3a), as well as liquid scintillation counting measurements (Fig. 3b). SCU-CPN-1 shows a surprisingly high removal rate for TcO_4_^−^ from aqueous solution. Instantly after the contact (<30 s, which is in fact required for the experimental operation), the characteristic peak of TcO_4_^−^ in the spectrum completely disappeared, representing the fastest TcO_4_^−^ sorption kinetics and shortest equilibrium time that has ever been reported for an anion-exchange material (Supplementary Table 1). The desorption of Cl^−^ into the solution was monitored by ion chromatograph to be as rapid as the TcO_4_^−^/ReO_4_^−^ uptake (Supplementary Fig. 6a), supporting the anion-exchange mechanism. The sorption kinetics of TcO_4_^−^ by Purolite A532E and A530E (Fig. 3b), which are regarded as standard commercial anion-exchange resins for efficiently removing TcO_4_^−^ from aqueous solution, were also tested under identical sorption conditions. In sharp contrast, SCU-CPN-1 removes more than 99% of TcO_4_^−^ from aqueous solution in less than 30 s, whereas Purolite A532E and A530E resins take at least 120 min to reach their sorption equilibrium and uptake only 32% and 38% of TcO_4_^−^ in 5 min, respectively. A further sorption experiment using Purolite A530E with a smaller particle size (<100 μm, denoted as Purolite A530E-ground) shows a much enhanced sorption kinetics, where the sorption equilibrium time is reduced to 5 min (Supplementary Fig. 6b). This suggests the small particle size of SCU-CPN-1 should be a major origin for the faster exchange kinetics observed. Although SCU-CPN-1 is non-porous for N_2_ uptake, it seems to be porous enough for rapid anion transportation during the exchange process. Comparison of crystalline cationic MOFs with ordered pore channels further highlights the advanced TcO_4_^−^ removal rate that SCU-CPN-1 exhibits. For example, the two most optimal cationic MOFs, SCU-100 and SCU-101 reported recently, take 30 and 10 min to reach their sorption equilibrium, respectively^19,21^. Such an advantage would be further beneficial by decreasing the radiation dose received during highly radioactive waste management. This also provides an extremely attractive emergency response material for efficiently controlling the accidental release of ^99^Tc into the environment, where the sorption rate is the most critical parameter. In order to analyze the sorption mechanism of SCU-CPN-1 in detail, we have investigated the sorption kinetics again by lowering the sorbent loading and ReO_4_^−^concentration (Supplementary Fig. 6c), showing a pseudo-second order reaction process (Supplementary Table 2). Under this condition, SCU-CPN-1 still performs much better than Purolite A530E-ground. SCU-CPN-1 reaches the sorption equilibrium in 10 min whereas it takes at least 140 min to reach the sorption equilibrium for Purolite A532E-ground. The rate constant of SCU-CPN-1 is much larger than that of Purolite A530E-ground. Additionally, the distribution coefficient (*K*_d_) of SCU-CPN-1 toward ReO_4_^−^ reaches as high as 6.2 × 10^5^ ml g^−1^, which is comparable to the highest reported value (Supplementary Table 3), showing promises in the removal depth for the environmental remediation applications.

### Sorption isotherm analysis

**Fig. 4:**
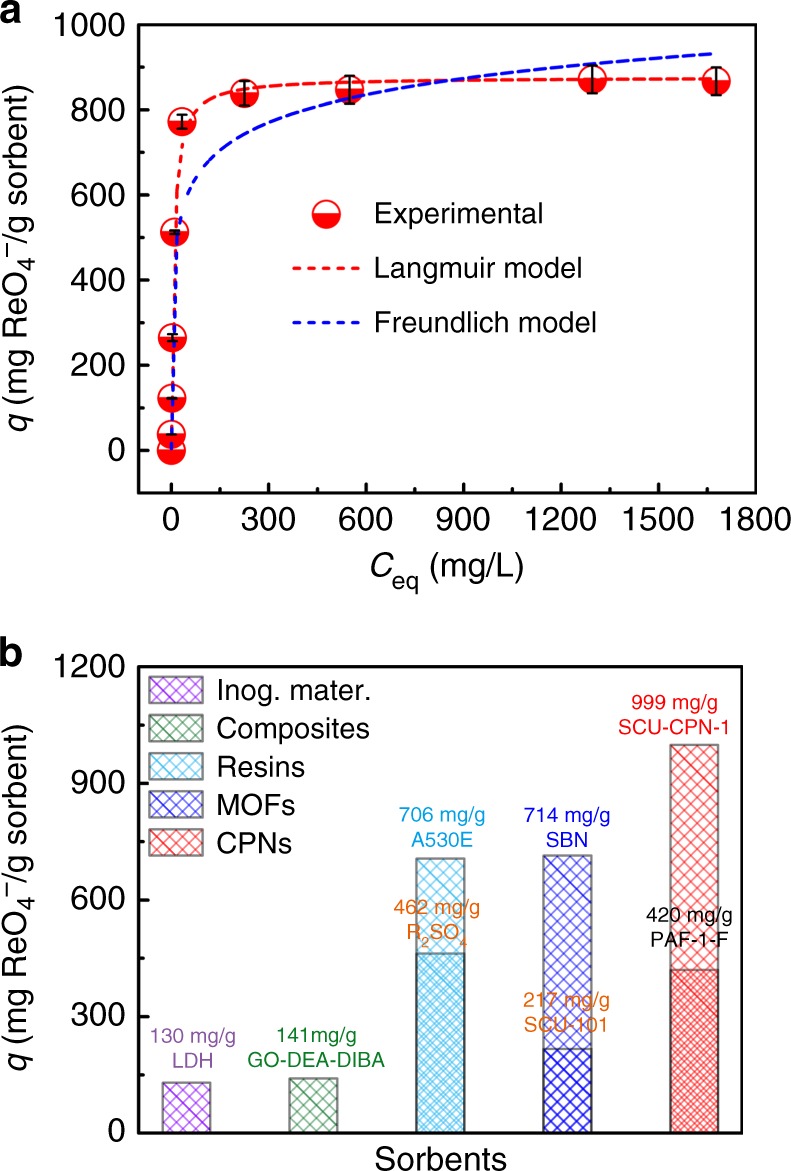
Sorption isotherm investigations of SCU-CPN-1. **a** Sorption isotherm of SCU-CPN-1 for ReO_4_^−^ uptake. **b** ReO_4_^−^ sorption capacity of SCU-CPN-1 compared with other reported anion sorbents. Error bars represent S.D. (standard deviation). *n* = 3 independent experiments

We further evaluated the sorption capacity of SCU-CPN-1 toward TcO_4_^−^. Due to the limited amount of ^99^Tc available, as well as the regulation on the amount of radioactive materials that can be handled, ReO_4_^−^ was used as a surrogate for TcO_4_^−^ given their almost identical charge densities and therefore chemical behaviors^44^. In fact, this was further confirmed based on the sorption kinetics data under the same sorption conditions (Fig. 3b). From the sorption isotherm curve (Fig. 4a), the saturated sorption capacity calculated from fitting based on Langmuir model (see details in SI) is 876 mg ReO_4_^−^ g^−1^ SCU-CPN-1 material (Supplementary Table 4). When a small excess of ReO_4_^−^ is present in solution, almost 100% of Cl^−^ anions in SCU-CPN-1 can be quantitatively replaced by ReO_4_^−^, as determined from ICP analysis of ReO_4_^−^ sorbed samples, giving a notably large exchange capacity of 999 ± 20 mg ReO_4_^−^ g^−1^ SCU-CPN-1 material (Supplementary Table 5), being very close to the theoretic sorption capacity of 1058 mg g^−1^ calculated by assuming the complete anion-exchange. This capacity is significantly larger than those of Purolite A532E (446 mg g^−1^) and A530E (706 mg g^−1^). As a comprehensive comparison, we summarize the TcO_4_^−^/ReO_4_^−^ sorbent materials into five different categories (Fig. 4b and Supplementary Table 6): (1) purely inorganic materials^14,17,18,45–48^, (2) composites^49–51^, (3) resins^13,52–55^, (4) MOFs^19–22,56–58^, and (5) CPNs^30^. Purely inorganic materials and composites exhibit the lowest sorption capabilities toward ReO_4_^−^, with maximum capacities reaching 130 and 141 mg g^−1^, respectively. The cationic MOF materials hold the maximum sorption capacity of 714 mg g^−1^, which is close to commercial anion-exchange resins. Banerjee et al^30^. reported a functionalized porous aromatic framework PAF-1-F with a ReO_4_^−^ sorption capacity of 420 mg g^−1^. In this work, SCU-CPN-1 sets a new capacity record for removing TcO_4_^−^/ReO_4_^−^.

### Stability under extreme conditions

**Fig. 5:**
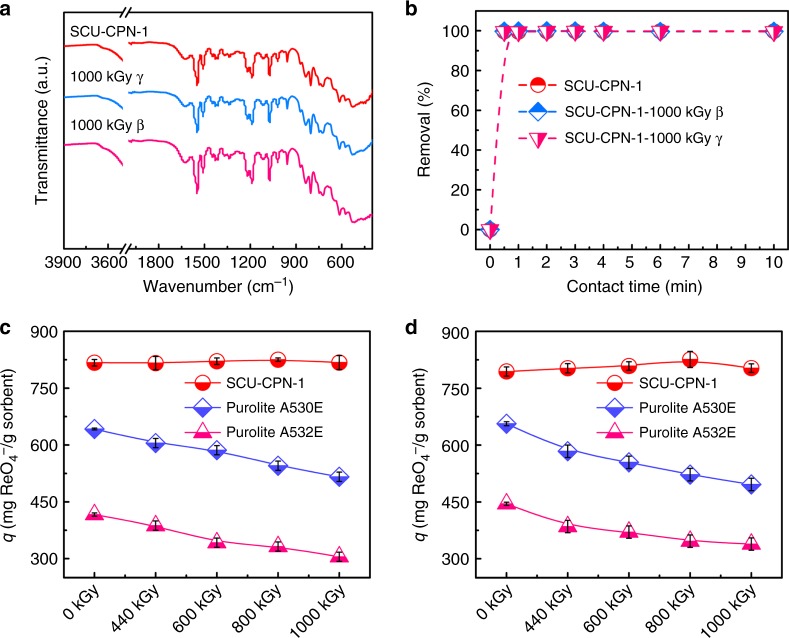
Radiation-resistance measurements of SCU-CPN-1. **a** FT-IR spectra of SCU-CPN-1 before and after being irradiated by 1000 kGy of β-rays or γ-rays. **b** Sorption kinetics of SCU-CPN-1 before and after irradiation with 1000 kGy of β-rays or γ-rays. **c** Sorption capacities of ReO_4_^−^ by SCU-CPN-1, Purolite A530E, and A532E after being irradiated with varied doses of β-rays. **d** Sorption capacities of ReO_4_^−^ by SCU-CPN-1, Purolite A530E, and 532E after being irradiated with varied doses of γ-rays. Error bars represent S.D. *n* = 3 independent experiments

More importantly, SCU-CPN-1 exhibits outstanding resistance toward high-energy ionizing radiation, including β-rays and γ-rays, providing a substantial prerequisite for application in used nuclear fuel reprocessing and waste management. The radiation-resistance is confirmed by SEM (Supplementary Fig. 7b, c), FT-IR spectra (Fig. 5a), sorption kinetics (Fig. 5b), and sorption capacity (Fig. 5c, d) measurements on the irradiated samples. Very impressively, the microsphere morphology, sorption rate and capacity for ReO_4_^−^ by SCU-CPN-1 remains unaffected by either radiation type, even after irradiation with extremely large doses of 1000 kGy of β-rays and γ-ray. In comparison, the sorption capacities of ReO_4_^−^ by Purolite A532E and A530E gradually decrease as the irradiation dose increases, consistent with the literature reports^59^. After receiving 1000 kGy of either β-rays or γ-rays, the sorption capacities exhibit an ~30% reduction for both resins (Fig. 5c, d), implying poor performance for treating used fuel solutions. This obvious difference originates from the conjugated-ring-rich nature of SCU-CPN-1 and more specifically that the key functional groups of cationic imidazolium are protected within a large conjugated system, while the quaternary ammonium salts in resin materials are easily subject to radiation-induced degradation.

**Fig. 6:**
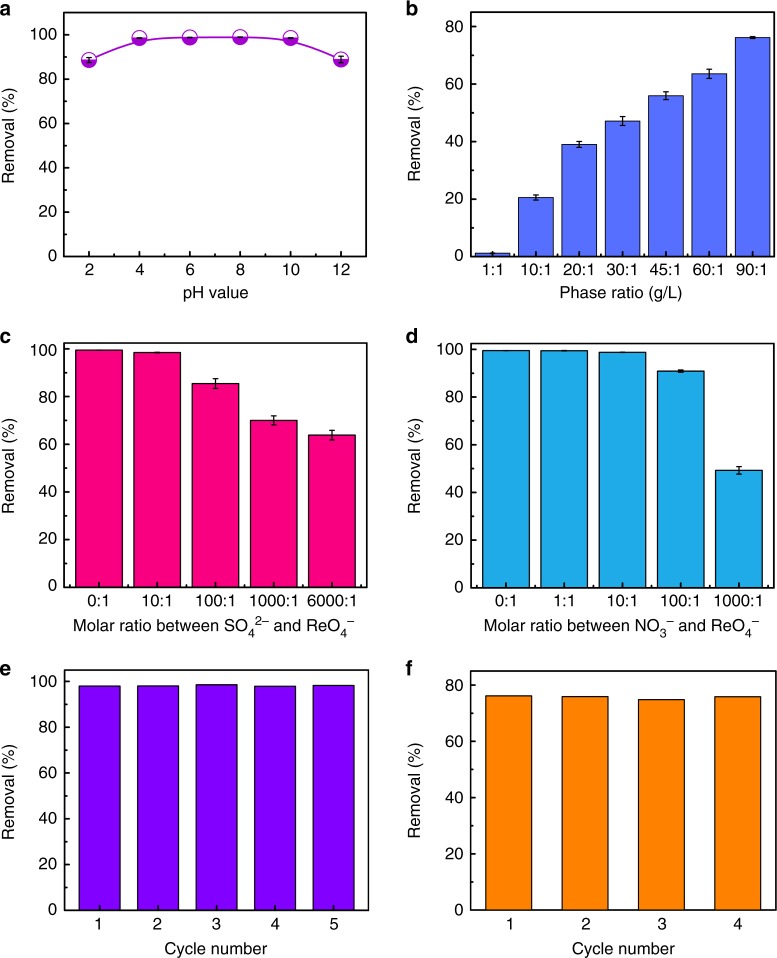
Acid stability, selectivity and reusability of SCU-CPN-1. **a** Effect of pH on the sorption properties of ReO_4_^−^ by SCU-CPN-1. **b** Removal of ReO_4_^−^ by SCU-CPN-1 as a function of solid/liquid ratio in 3 M HNO_3_ solution. **c** Effect of competing SO_4_^2−^ anions on the anion-exchange of ReO_4_^−^ by SCU-CPN-1. **d** Effect of competing NO_3_^−^ anions on the anion-exchange of ReO_4_^−^ by SCU-CPN-1. **e** Reversibility of SCU-CPN-1 for removing ReO_4_^−^ at pH 7. **f** Reversibility of SCU-CPN-1 for removing ReO_4_^−^ under the condition of 3 M HNO_3_. Error bars represent S.D. *n* = 3 independent experiments

Furthermore, the ReO_4_^−^ uptake capacity of SCU-CPN-1 remains almost unchanged over a wide pH range from 2 to 12 (Fig. 6a), initially indicating a good uptake selectivity and hydrolytic stability. The morphology and the characteristic peak of SCU-CNP-1 (1074 cm^−1^) in the FT-IR spectrum remain unchanged after being immersed in 3 M HNO_3_ solution for 12 h (Supplementary Fig. 8), consistent with the proposed acidic stability, which can be further confirmed by the material regeneration test discussed below.

### Uptake selectivity

In highly radioactive wastes, a large excess of competing anions, such as NO_3_^−^ and SO_4_^2−^, often coexist with TcO_4_^−^. NO_3_^−^ is present in the highly acidic solution (e.g., 3 M HNO_3_) of used fuel and SO_4_^2−^ is often included in the high-level liquid waste. We therefore measured the sorption properties of ReO_4_^−^ by SCU-CPN-1 as a function of NO_3_^−^ and SO_4_^2−^ concentration. The results show that the removal of ReO_4_^−^ occurs at 91% when the molar ratio of NO_3_^−^ and ReO_4_^−^ is as high as 100:1 (Fig. 6d). We further prepared a 3 M HNO_3_ solution containing 343 ppm of ReO_4_^−^ (NO_3_^−^:ReO_4_^−^ molar ratio = 2186). It should be noted that there is no reported material that can remove ReO_4_^−^/TcO_4_^−^ under this condition. Impressively, SCU-CPN-1 can extract approximately 40% of ReO_4_^−^ at a solid/liquid ratio of 20. When the solid/liquid ratio was increased to 90 (a number that is relevant to the chromatographic column application), SCU-CPN-1 is able to remove 76% of available ReO_4_^−^ from solution (Fig. 6b). This feature clearly represents a priority in uptake for ReO_4_^−^/TcO_4_^−^ over NO_3_^−^, and attains to one of the most crucial aspects for removing TcO_4_^−^ from reprocessed used fuel prior to PUREX. Note this is unattainable for any of the cationic MOFs or purely inorganic materials, which are either instable or completely lose their uptake capability under highly acidic condition. Moreover, with the coexistence of a large excess of SO_4_^2−^ with a much higher charge density, SCU-CPN-1 still extracts 85% and 64% of available ReO_4_^−^ when the SO_4_^2−^:ReO_4_^−^ molar ratio are 100:1 and 6000:1, respectively (Fig. 6c). This excellent selectivity is surprising because high-charge anions often outcompete with low-charge anions during the anion-exchange process given their stronger electrostatic interaction with the host materials^18,30^. Likely, it can be ascribed to the hydrophobic nature of the pores that possess a higher affinity for those anions with lower charge densities, similar with traditional anion-exchange resins and cationic MOF systems^19–22^.

### Removal from simulated nuclear wastes

Because critical assessment of the sorption kinetics and radiation stability revealed several superior features combined into one material, the next logical step was to test the capabilities that SCU-CPN-1 has when separating TcO_4_^−^ in simulated nuclear waste solutions. In the Hanford LAW stream (Supplementary Table 7), the amounts of NO_3_^−^, NO_2_^−^, and Cl^−^ far exceed that of TcO_4_^−^ by more than 300 times. Following similar experimental conditions as we have used earlier, SCU-CPN-1 was found to sequester almost 90% of available TcO_4_^−^ from the waste in a solid/liquid ratio of 5 (Supplementary Table 8), which is notably better than the removal using SCU-101 (75.2% at a solid/liquid ratio of 10) and NDTB-1 (13% at a solid/liquid ratio of 5) that have been previously studied^17–19^.

### Material recyclability

SCU-CPN-1 is also a completely reversible anion-exchange material and the microsphere morphology remains unchanged after anion-exchange (Supplementary Fig. 7a), making it very cost-effective for separation applications. More than 97% of initially sorbed ReO_4_^−^ in SCU-CPN-1 could be exchanged with Cl^−^ or NO_3_^−^ in 1 M NaCl or NaNO_3_ solutions heated at 80 °C. (Supplementary Table 9). The elevated temperature required for the complete desorption also indicates a strong interaction between SCU-CPN-1 and TcO_4_^−^/ReO_4_^−^, which is responsible for the excellent TcO_4_^−^ uptake capability, especially the selectivity. Even after five sorption/desorption cycles, SCU-CPN-1 remains stable and the sorption properties are not influenced (Fig. 6e). More impressively, under the condition of 3 M HNO_3_, SCU-CPN-1 remains reusable and the sorption properties are unaffected after at least four runs (Fig. 6f), indicating this material is amazingly stable and fully recyclable under the real used fuel repossessing condition.

### Sorption mechanism

**Fig. 7:**
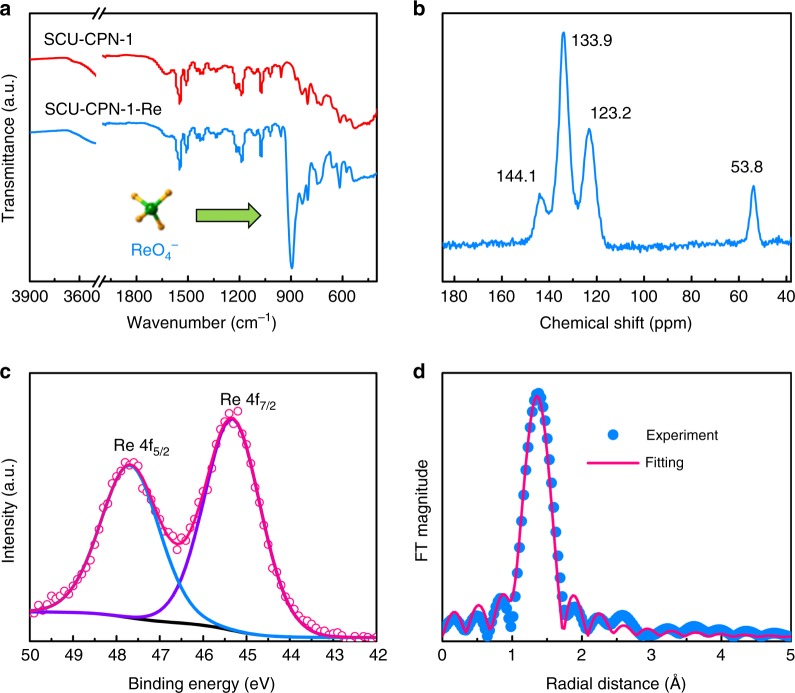
FT-IR, solid-state ^13^C NMR, XPS, and EXAF spectra of SCU-CPN-1-Re. **a** FT-IR spectra of SCU-CPN-1 before and after anion-exchange with ReO_4_^−^. **b** Solid-state ^13^C NMR spectrum of SCU-CPN-1 after anion-exchange with ReO_4_^−^. **c** XPS analysis of Re 4*f* from ReO_4_^−^ sorbed SCU-CPN-1. **d** *k*^3^-weighted Re L_III_-edge EXAFS spectra of ReO_4_^−^ sorbed SCU-CPN-1 sample. Dots: experimental data; solid lines: fitted data

The anion-exchange process was further confirmed by TEM-EDS (Fig. 1c) and supported by the evolution of a new peak at 896 cm^−1^ (Re–O ν_3_ asymmetric stretch) in the FT-IR spectra after anion-exchange with ReO_4_^−^ (Fig. 7a). In the solid-state ^13^C NMR spectrum of SCU-CPN-1-Re, the peak feature for carbon atoms bound to the nitrogen atom shifts from 142.9 to 144.1 ppm after ReO_4_^−^ uptake, while other signals remained unchanged. This result hints for the interaction between ReO_4_^−^ with the cationic imidazole group in SCU-CPN-1 (Fig. 7b), further confirmed by the theoretical study discussed below. In addition, extended X-ray absorption fine structure (EXAFS) were carried out to investigate the local environment of ReO_4_^−^ in SCU-CPN-1. The EXAFS spectrum of SCU-CPN-1-Re sample consists of a Re–O shell at a bond distance (*R*) of 1.73 Å with a coordination number (*N*) of 4.1 (Fig. 7d and Supplementary Table 10), very similar to those of the reference sample, NaReO_4_ (*R* = 1.74 Å, *N* = 4)^60^. This indicates that the Re species in SCU-CPN-1-Re exist as perrhenate, keeping the oxidation state of Re(VII), consistent with the XPS analysis result (Fig. 7c).

**Fig. 8:**
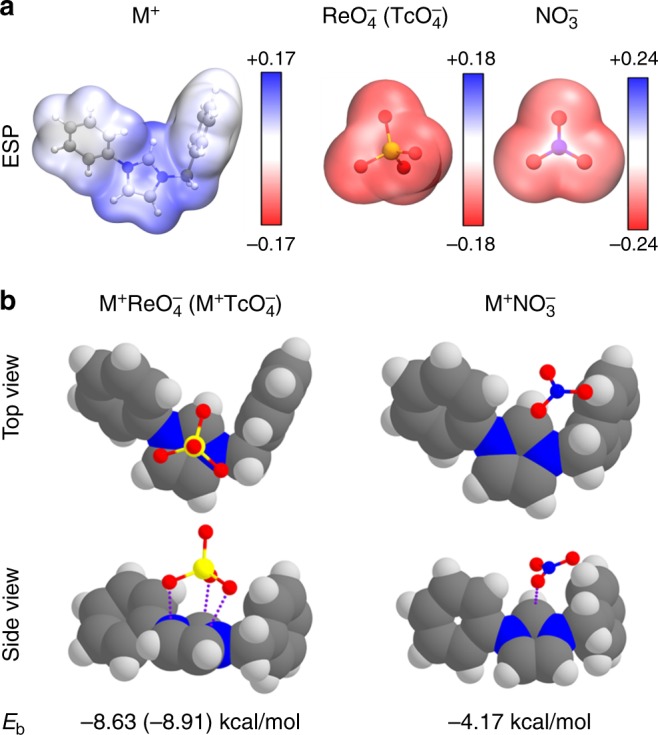
Density functional theory analysis. **a** Electrostatic potential (ESP) distributions on the van der Waals surfaces (isodensity = 0.001 a.u.) of M^+^ ([C_6_H_5_–C_3_N_2_H_3_–CH_2_–C_6_H_5_]^+^ fragment), ReO_4_^−^ (TcO_4_^−^) and NO_3_^−^. **b** Optimized sorption complexes of M^+^ReO_4_^−^ (M^+^TcO_4_^−^) and M^+^NO_3_^−^. The structures of M^+^ReO_4_^−^ and M^+^TcO_4_^−^ are almost identical. Binding energies (*E*_b_) between M^+^ and anions are labeled below each complex

To unravel the intrinsic driving force for the superior TcO_4_^−^ separation capabilities by SCU-CPN-1, we performed DFT theoretical studies aimed at analyzing the interaction between the repeating unit as a fragment of SCU-CPN-1 and TcO_4_^−^/ReO_4_^−^. We also made a comparison with the case of NO_3_^−^ in order to elucidate the origin of uptake selectivity and the surprising TcO_4_^−^/ReO_4_^−^ removal capability from highly acidic conditions. The framework of our synthetic SCU-CPN-1 material possesses large positive charges since each N3 atom of the imidazole ring should formally be +1 charged when connecting a methylene. DFT calculations on a local fragment of the material, M^+^ ([C_6_H_5_–C_3_N_2_H_3_–CH_2_–C_6_H_5_]^+^ fragment), show that the positive ESP (blue areas) mainly distributes on the vdW surface and delocalizes on the imidazole ring, propagating on the N1, C2, N3 atoms and three bounded H atoms, as shown in Fig. 8a. The C4 and C5 atoms do not contribute obviously to the positive ESP. In contrast, ESP distributed on the vdW surfaces of all anions are negative (red areas). Therefore, the local imidazole ring of the material should have a crucial role for attracting these anions by strong electrostatic interaction. Figure 8b shows the stable sorption structures of M^+^ReO_4_^−^ (M^+^TcO_4_^−^) and M^+^NO_3_^−^. It can be seen that one side of the ReO_4_^−^(TcO_4_^−^) tetrahedron, consisting of three oxygen atoms, is almost parallel to the imidazole ring, forming a face-to-face stacking structure. However, NO_3_^−^ (as well as Cl^−^) is more inclined to be close to the C2 atom, creating a corner-to-corner interaction. The two distinctly different structures result in the binding energies in M^+^ReO_4_^−^ (-8.63 kcal mol^−1^) and M^+^TcO_4_^−^ (−8.91 kcal mol^−1^), being higher than twice of that for M^+^NO_3_^−^ (−4.17 kcal mol^−1^).

**Fig. 9:**
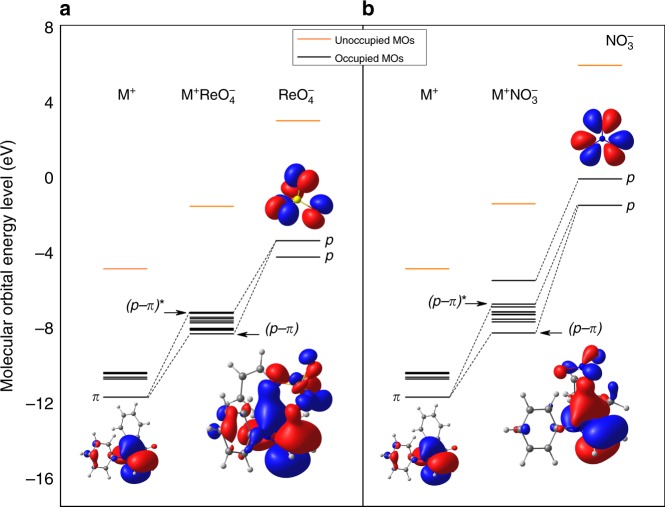
Molecular orbital interaction diagrams. Molecular orbital interaction diagrams of **a** M^+^ReO_4_^−^ and **b** M^+^NO_3_^−^

The different sorption structure between M^+^ReO_4_^−^(M^+^TcO_4_^−^) and M^+^NO_3_^−^ can be further understood by investigating their electronic structures. As discussed above, the C1, N2, and C3 atoms attract one O atom of the anions through strong electrostatic interactions. Furthermore, molecular orbital analyses show that the N1–C2–N3 *π* bond hybridize with the *p* lone pair electrons of ReO_4_^−^ (Fig. 9a), forming relatively strong *p-π* interactions that leads to the face-to-face stacking M^+^ReO_4_^−^ structure. Meanwhile, although the C4 and C5 atoms of the imidazole ring do not contribute to the positive ESP, the C4–C5 *π* bond could also hybridize with the *p* lone pair electrons of another O atom of ReO_4_^−^ simultaneously (Fig. 9a). In contrast, for NO_3_^−^ (as well as Cl^−^), the *p* lone pair electrons of only one O atom can hybridize with the N1–C2–N3 *π* bond of the of the imidazole ring (Fig. 9b). These results suggest cationic imidazole ring is the key functional group for the selective uptake of TcO_4_^−^/ReO_4_^−^ over NO_3_^−^, which should be further considered for anion-exchange material design in the future.

## Discussion

In conclusion, we document here our very recent discovery on the significant TcO_4_^−^ separation capabilities of a cationic polymeric network material (SCU-CPN-1). SCU-CPN-1 has the potential to be applied in many areas, including used nuclear fuel reprocessing prior to PUREX separations, nuclear waste management ahead of vitrification, remediation of ^99^Tc contamination in the environment, and emergency response in the event of a nuclear accident. Not only does SCU-CPN-1 set new records for TcO_4_^−^/ReO_4_^−^ uptake kinetics and sorption capacity, but it also exhibits excellent uptake performances from highly acidic solutions even in the presence of large excesses of other competing anions. The decent radiation-resistance observed in this material, which is atypical for traditional anion-exchange resins, coupled with the ability to be proficiently recycled make these potential applications more practical and cost-effective. Furthermore, the NO_3_^−^ exchanged version of SCU-CPN-1 is fully combustible, meeting the CHON-only standard. Such a synthetic and design strategy can be certainly expanded for the separation of actinides (U, Np, Pu, Am, etc.) and other types of fission products. The foregoing results demonstrate that the family of CPN materials show powerful application potentials in the nuclear industry and their utilities may be realized in the near future.

## Methods

### Safety note

Caution! Although Tc-99 is a low-energy β-emitter (*t*_1/2_ = 2.13 × 10^5^ a), it still possesses significant health risks when inhaled or digested. Standard precautions and procedures for handling radioactive materials should be followed, and all Tc-99 studies were conducted in a licensed laboratory dedicated to radiological investigations.

### Chemicals and reagents

1,1,2,2-tetrakis(4-(imidazolyl-4-yl)phenyl)ethene (TIPE) and 1,4-bis(bromomethyl)benzene (BBB) were provided by Beijing HWRK Chem Co., LTD and J&K Scientific LTD, respectively. Commercial anion-exchange resins, Purolite A530E and A532E, were provided from Purolite Co., Ltd, and dried at 60 °C for 24 h before use. NaReO_4_, HCl, NaOH, and other reagents were analytically pure and used as received. ^99^TcO_4_^−^ stock solutions were prepared by dissolving desired amounts of NH_4_TcO_4_ (99%) solid in deionized water.

### Physical property measurements

A Thermo Nicolet iS50 spectrometer was used to collect the FT-IR spectra in the range of 4000–400 cm^−1^. Powder X-ray diffraction (PXRD) patterns were collected from 5° to 50° on a Bruker D8 Advance diffractometer equipped with Cu Kα radiation (*λ* = 1.54056 Å) and a Lynxeye one-dimensional detector. Thermogravimetric analysis was carried out in the range of 30−600 °C under a nitrogen flow with a heating rate of 10 °C min^−1^ on a NETZSCH STA 449F3 instrument. SEM and energy-dispersive spectroscopy (EDS) images were acquired with the energy of the electron beam being 20 keV using a FEI Quanta 200FEG scanning electron microscope (SEM). The morphology and elemental mapping were recorded by a FEI Tecnai G2 field emission high-resolution transmission electron microscope (HRTEM) with an accelerating voltage of 200 kV. The solid-state ^13^C NMR spectroscopy was performed on a Bruker Avance III 400 HD instrument with a Larmor frequency of 100.65 MHz. The concentrations of TcO_4_^−^ in solution were measured using a UV–vis spectrometer (Varian Cary 6000i) by monitoring the characteristic absorption peak at 290 nm and the activity of ^99^TcO_4_^−^ was also analyzed by a liquid scintillation counting (LSC) system (Perkin Elmer Quantulus 1220). The concentrations of ReO_4_^−^ in solution were measured by inductively coupled plasma-atomic emission spectrometry (ICP-AES, Thermo Fisher Scientific iCAP 7000). The concentrations of Cl^−^ in solution as the function of sorption time were evaluated by a 930 Compact IC Flex ion chromatograph instrument (Metrohm, Switzerland). Rhenium L_III_-edge EXAFS spectra were collected at the Shanghai Synchrotron Radiation Facility (SSRF, BL14W, Shanghai, China) using a silicon (111) double-crystal monochromator in fluorescence mode. The analyses and fitting of the EXAFS data were performed using the Athena and Artemis packages of the IFEFFIT 7.0 software. The surface state and electronic structure of the material were obtained by X-ray photoelectron spectroscopy (XPS) measurement (SPECS Corporation), equipped with a monochromator delivering Al Kα radiation (1486.7 eV) as the excitation source. The Re 4*f* XPS spectra of SCU-CPN-Re fitting was carried out via Casa XPS software and the energy scale of the high-resolution spectra were calibrated by C1*s* peak at 284.8 eV.

### Synthesis of SCU-CPN-1

Overall, 0.134 g (0.225 mmol) of TIPE and 0.119 g (0.45 mmol) of BBB were added to 16 ml *N*,*N*-dimethyl formamide (DMF) in a 35 ml microwave tube (9.0 cm long cylindrical glass tube with the inside diameter of 2.5 cm and the external diameter of 3 cm). The resulting mixture was stirred at 100 °C for 4 h under the pressure of 150 psi and 150 W of power on a microwave synthesis system (CEM DISCOVER S). SCU-CPN-1-Br powders were obtained after centrifugation, then washed three times with DMF, ethanol, and deionized water each, followed by freeze-drying. After soaking the SCU-CPN-1-Br in saturated sodium chloride (NaCl) solution for 12 h, a yellow powder of SCU-CPN-1-Cl was obtained (yield: 61%).

### Batch experiments

All the experiments were conducted at 25 °C using the batch sorption method. The pH was adjusted to the desired value with NaOH and/or HNO_3_ solutions in 5 min. In a typical batch experiment, 10 mg of SCU-CPN-1 was placed into 10 ml of aqueous solution containing various concentrations of ReO_4_^−^/TcO_4_^−^. After being stirred at a rate of 120 rpm for a desired contact time, the samples were separated with a 0.22 μm nylon membrane filter (SANJIA Biochemical Supplies).

### Sorption kinetics study

The experiments were carried out at pH 7 with a solid/liquid ratio of 1 g l^−1^. In each sample, 10 mg of sorbent material (SCU-CPN-1, Purolite A530E, or A532E) was added to 10 ml of aqueous solution containing 28 ppm TcO_4_^−^. The samples were collected after stirring the mixture at a rate of 120 rpm for 30 s, 3 min, 10 min, 30 min, 60 min, and 90 min, respectively. As a comparison, the uptake kinetics experiment was then repeated by using the same molar amount of ReO_4_^−^ as the surrogate for TcO_4_^−^. Besides, the concentration of Cl^−^ during the ion exchange process was measured as a function of time by ion chromatograph method. In order to evaluate the influence of the particle size in sorption kinetics analysis, the Purolite A530E was ground in an agate mortar and filtered using a 0.106 mm sieve and the particle size was further characterized by SEM image (Supplementary Fig. 9). The sorption kinetics of ground version of Purolite A530E was analyzed under the same uptake condition.

In order to study the sorption mechanism of SCU-CPN-1, 25 mg of SCU-CPN-1 was added to 500 ml of aqueous solution containing 14 ppm ReO_4_^−^. 1 ml of samples were taken after stirring the mixture at a rate of 120 rpm for 30 s, 1 min, 2 min, 3 min, 4 min, 5 min, 7.5 min, 10 min, 15 min, 30 min, 60 min, 90 min, and 110 min, respectively. The samples were separated using a 0.22 µm nylon membrane filter for ICP analysis. The uptake kinetics experiment was then repeated using Purolite A530E-ground for comparison.

Two kinetic models, including pseudo-first-order model (1) and pseudo-second-order model (2) were used to fit the sorption kinetics data. These two models can be expressed in the following equations:

1 $${\mathrm{Pseudo\operatorname{-}first}}\;{\mathrm{order}}\;{\mathrm{model}}:\;{\mathrm{ln}}(q_{\mathrm{e}} - q_t) = {\mathrm{ln}}\;q_{\mathrm{e}} - k_1t$$

2 $${\mathrm{Pseudo\operatorname{-}second}}\;{\mathrm{order}}\;{\mathrm{model}}:\frac{t}{{q_t}} = \frac{1}{{k_2q_{\mathrm{e}}^2}} + \frac{t}{{q_{\mathrm{e}}}}$$

where *k*_1_ and *k*_2_ (g mg^−1^ min^−1^) are the rate constants of pseudo-first-order and pseudo-second-order, respectively. *q*_*t*_ is the sorption capacity at contact time *t* and can be calculated by the following equation: $q_t = \frac{{\left( {C_0 - C_t} \right)V}}{m}$, where *m* and *V* are the mass of sorbent and solution volume, respectively. *C*_0_ and *C*_*t*_ are the concentration of ReO_4_^−^/TcO_4_^−^ at initial time and time *t*, respectively. The linearized plot of pseudo-first-order model and pseudo-second-order model was obtained when we plotted ${\mathrm{ln}}(q_{\mathrm{e}} - q_t)$ and *t/q*_*t*_ against *t*, respectively.

The distribution coefficient (*K*_d_) of SCU-CPN-1 toward ReO_4_^−^ was calculated by the following equation: $K_{\mathrm{d}} = \frac{{\left( {C_0 - C_{\mathrm{e}}} \right)V}}{{mC_{\mathrm{e}}}}$, where *C*_e_ is the equilibrium concentration of ReO_4_^−^/TcO_4_^−^.

### Sorption isotherm investigations

The sorption isotherm experiments of SCU-CPN-1 were conducted by varying the initial concentrations of ReO_4_^−^ ranging from 10 to 2500 ppm. 10 mg of SCU-CPN-1 was added into 10 ml of ReO_4_^−^ aqueous solution at pH 7 with the solid/liquid ratio of 1 g l^−1^. After being stirred at a rate of 120 rpm for 12 h, the suspension was separated with a 0.22 µm nylon membrane filter for ICP analysis. Sorption isotherms experiments of resins (Purolite A530E and A532E) were also carried out for comparison.

The Langmuir isotherm is based on the assumption that the sorption is monolayer sorption and all sites were equal. The linear equation of the Langmuir isotherm model is expressed as followed^61^:

3 $$\frac{{C_{\mathrm{e}}}}{{q_{\mathrm{e}}}} = \frac{1}{{q_{\mathrm{m}}K_{\mathrm{L}}}} + \frac{{C_{\mathrm{e}}}}{{q_{\mathrm{m}}}}$$

where *q*_m_ (mg g^−1^) is the maximum sorption capacity of Re(VII) corresponding to complete monolayer coverage and *K*_L_ (l mg^−1^) is a constant indirectly related to sorption capacity and energy of sorption, which characterizes the affinity of the adsorbate with the adsorbent. *K*_L_ is a constant of Langmuir model.

The Freundlich equation is an empirical equation based on sorption on a heterogeneous surface. The stronger binding sites are occupied first and with the increase of site occupation the binding strengths are decreased. The linear equation can be expressed by:

4 $${\mathrm{ln}}q_{\mathrm{e}} = {\mathrm{ln}}\;K_{\mathrm{F}} + \frac{1}{n}{\mathrm{ln}}\;C_{\mathrm{e}}$$

where *K*_F_ and *n* are the Freundlich constants related to the sorption capacity and the sorption intensity, respectively.

### pH effect study

The effect of pH value for ReO_4_^−^ sorption was carried out by ranging pH values from 2 to 12. 10 mg of SCU-CPN-1 was added to 10 ml of aqueous solution containing 400 ppm ReO_4_^−^. After being stirred at a rate of 120 rpm for 12 h, the resulting mixture was separated with a 0.22 µm nylon membrane filter for ICP analysis.

### Anion-exchange selectivity study

The effect of competing ions was studied by adding different concentration of NaNO_3_ (0.15 mM, 1.5 mM, 15 mM, and 150 mM) or Na_2_SO_4_ (0.015 mM, 0.15 mM, 1.5 mM, 15 mM, 150 mM, and 900 mM) into a 0.15 mM ReO_4_^−^solution, respectively. After being stirred at a rate of 120 rpm for 12 h, the suspension was separated with a 0.22 µm nylon membrane filter for ICP analysis.

### Exchange experiments with simulated wastes

The simulated Hanford Low Activity Waste (LAW) Melter Recycle Stream was prepared according to a reported protocol^18^. Overall, 10 mg of SCU-CPN-1 was added to 10 ml of the above simulated wastes. After being stirred at a rate of 120 rpm for 12 h, the suspension was separated with a 0.22 µm nylon membrane filter for LSC analysis.

### Batch experiments in 3 M HNO_3_ system

1 ml of aqueous solution containing 343 ppm ReO_4_^−^ with 3 M HNO_3_ was mixed with 1 mg, 10 mg, 20 mg, 30 mg, 45 mg, 60 mg, and 90 mg of SCU-CPN-1-NO_3_ (SCU-CPN-1-NO_3_ was obtained by soaking SCU-CPN-1 in sat. NaNO_3_ solution for 12 h), respectively. After being stirred at a rate of 120 rpm for 12 h, the suspension was separated with a 0.22 µm nylon membrane filter for ICP analysis.

### Reusability study

After SCU-CPN-1 was sorbed with 28 ppm of ReO_4_^−^at pH 7, 10 mg of ReO_4_^−^-sorbed material was eluted by 10 ml of various concentrations of NaCl solution at 25 °C, 60 °C, and 80 °C to search for the optimum desorption condition. 150 mg of SCU-CPN-1 was first sorbed with 150 ml 28 ppm of ReO_4_^−^at pH 7, and then the ReO_4_^−^-sorbed material was eluted by 150 ml of 1 mol l^−1^ NaCl solution at 80 °C for 12 h followed by washing with distilled water for three times.

### Radiation-resistance measurements

A β irradiation experiment was conducted by irradiating SCU-CPN-1 at a dose rate of 50 kGy h^−1^ for four different doses (400, 600, 800, and 1000 kGy) and the source of β-ray was provided by an electron accelerator equipped with an electron beam (10 MeV). The γ-ray irradiation was provided by a ^60^Co irradiation source (92.42 PBq). SCU-CPN-1 was irradiated at a dose rate of 3.125 kGy h^−1^ or four different doses (400, 600, 800, and 1000 kGy), respectively. The radiation-resistance of SCU-CPN-1 was characterized by FT-IR spectroscopy and further checked by ReO_4_^−^ uptake kinetics and capacity experiments measured on the irradiated samples.

### Computational method

A [C_6_H_5_–C_3_N_2_H_3_–CH_2_–C_6_H_5_]^+^ fragment (M^+^ for short in the following, as shown in Supplementary Fig. 10) was used as the model for mimicking the positively charged local structure of the amorphous sorbent material of SCU-CPN-1. Four anions, including ReO_4_^−^, TcO_4_^−^, NO_3_^−^, and Cl^−^ were used as the sorbates. The isolated structures of each anion and the complexes of M^+^A^−^ (A^−^ = ReO_4_^−^, TcO_4_^−^, NO_3_^−^ and Cl^−^) were fully optimized in the gaseous phase using the B3LYP-D3/SDD~6-31G*^62–65^. For each complex, we considered various binding sites and the stable structures with lowest total energies are shown in Fig. 8. Higher precision single-point energy calculations were performed for these optimized structures in liquid phase (water as the solvent) at the M06-2×-D3/SDD~6-311++G**^62,65–68^ level. The SMD implicit solvent model was employed to consider the solvent effect. Based on the single-point energy calculations, the binding energies, *E*_b_, were calculated as

$$E_{\mathrm{b}} = E\left( {{\mathrm{complex}}} \right)-E({\mathrm{M}}^ + )-E\left( {{\mathrm{anion}}} \right)$$

where *E*(complex), *E*(M^+^), and *E*(anion) indicate the total energies of each complex, M^+^, and anions, respectively. All calculations were performed using the Gaussian 09 program^69^.

### Data availability

All data are either provided in the Article and its Supplementary Information or are available from the corresponding author upon request.

## Electronic supplementary material

Supplementary Information
