# Supplementary material for: 99TcO4− remediation by a cationic polymeric network
Source: Nat Commun. 2018 Aug 1;9:3007. doi: 10.1038/s41467-018-05380-5 (PMC6070552; doi:10.1038/s41467-018-05380-5)
Supplement: Supplementary file 1 — Supplementary Information [file 41467_2018_5380_MOESM1_ESM.pdf]

Supplementary Information

$^{99}TcO_4^-$ Remediation by a Cationic Polymeric Network

Li et al.

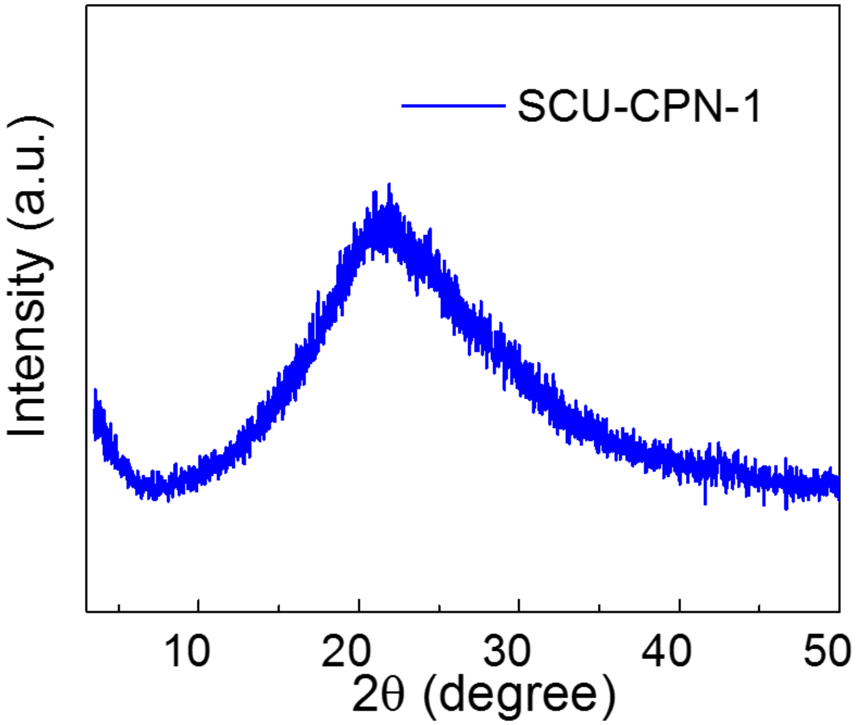

Supplementary Figure 1. PXRD data of SCU-CPN-1.

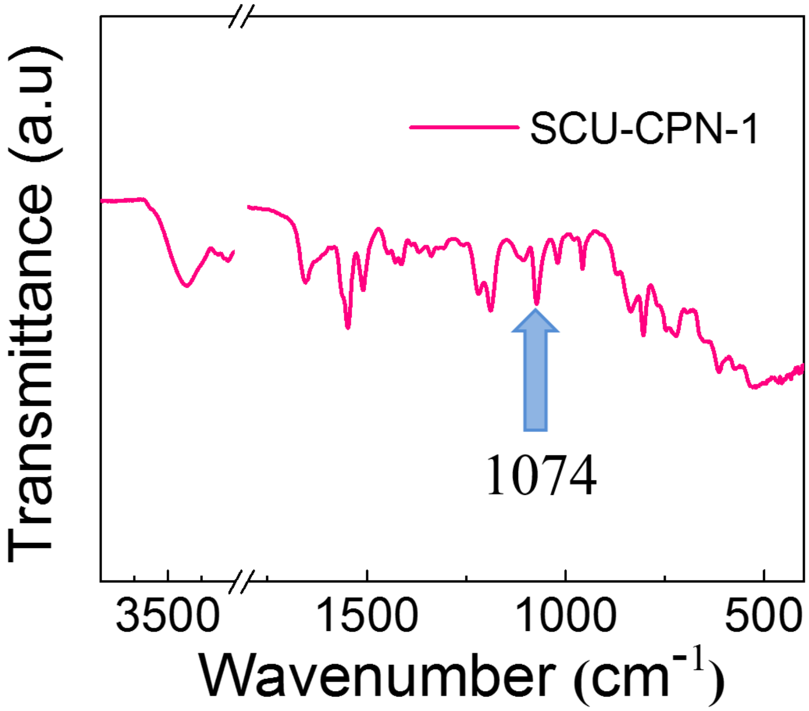

Supplementary Figure 2. FT-IR spectrum of SCU-CPN-1.

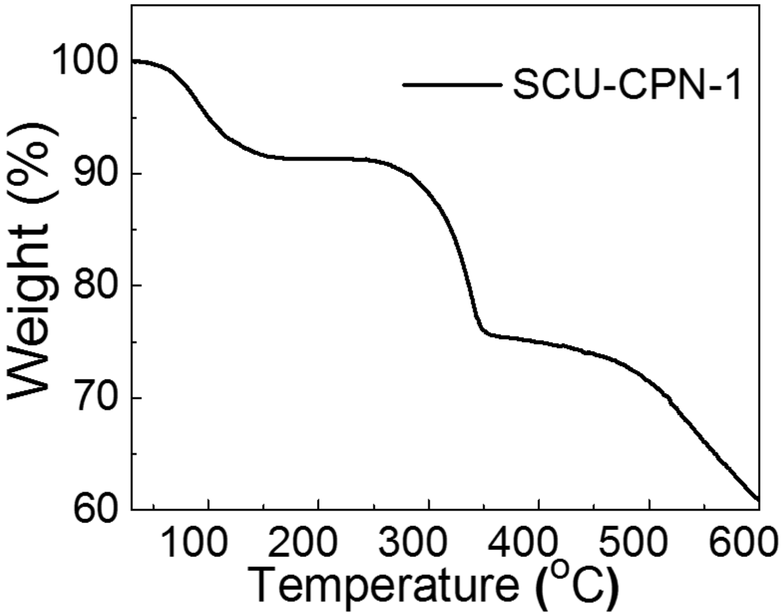

Supplementary Figure 3. TGA curves of SCU-CPN-1.

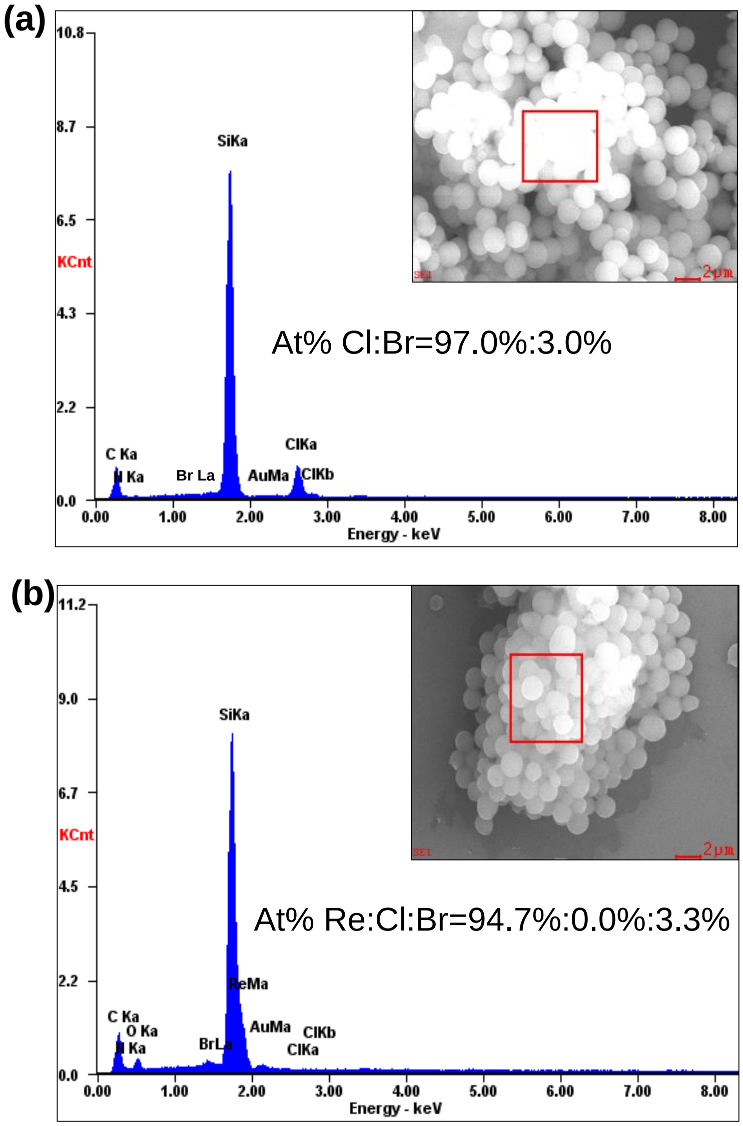

Supplementary Figure 4. SEM-EDS images of (a) SCU-CPN-1-Cl and (b) SCU-CPN-1-Re.

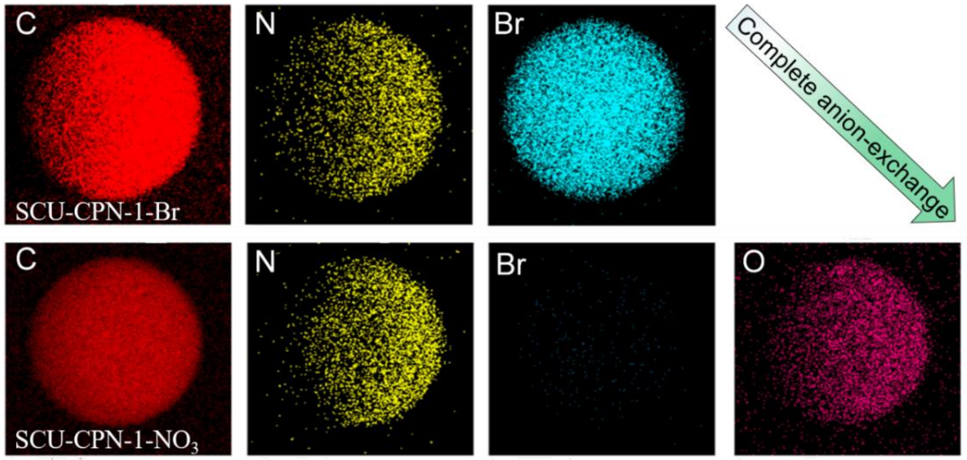

Supplementary Figure 5. EDS mapping of SCU-CPN-1-Br and SCU-CPN-1-NO_3.

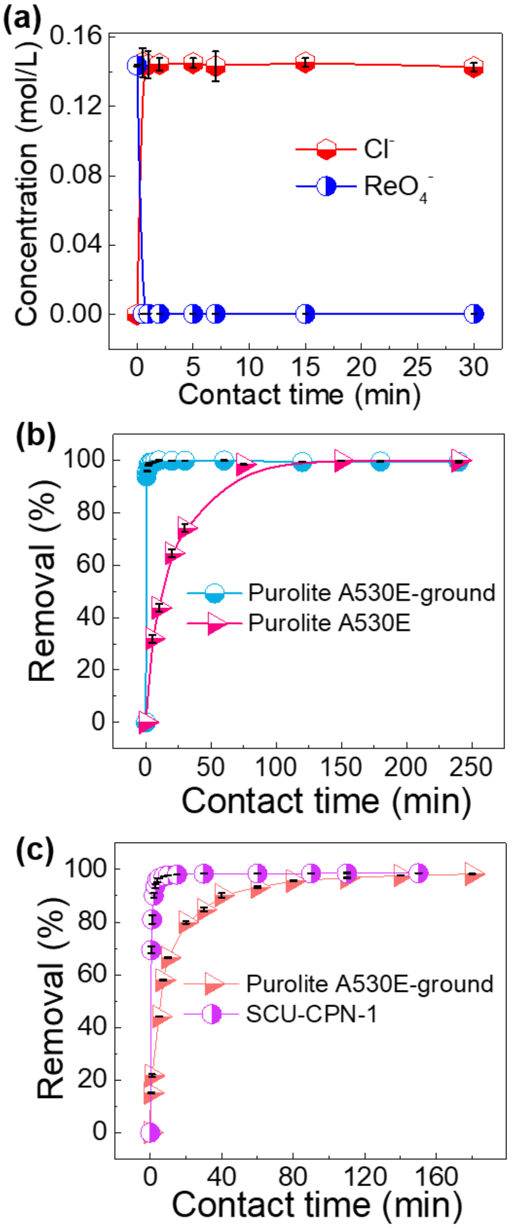

Supplementary Figure 6. Sorption kinetics of Purolite A530E-ground and SCU-CPN-1. (a) The concentration of Cl^- and ReO_4^- during the ion-exchange process (solid/liquid ratio of 1 g l^{-1} and 28 ppm of ReO_4^-). (b) Sorption kinetics of Purolite A530E and Purolite A530E-ground under the condition of solid/liquid ratio of 1 g l^{-1} and 28 ppm of ReO_4^-. (c) Sorption kinetics of SCU-CPN-1 and Purolite A530E-ground under the condition of solid/liquid ratio of 0.05 g l^{-1} and 14 ppm of ReO_4^-. Error bars represent S. D., n = 3 independent experiments.

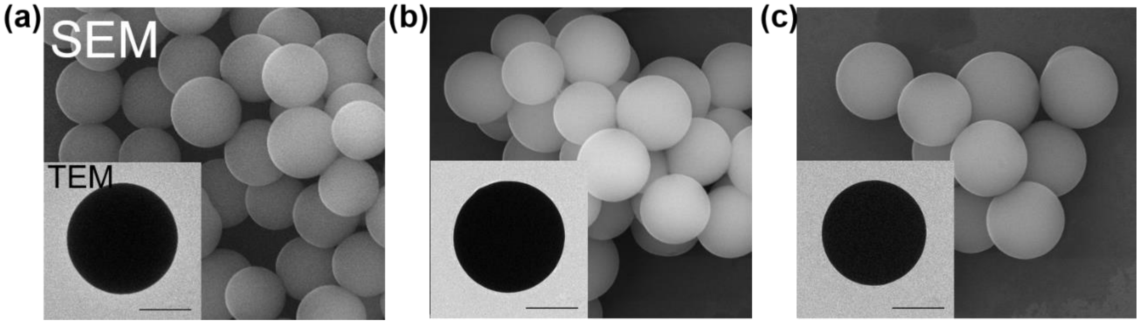

Supplementary Figure 7. SEM and TEM image of the materials. SEM and TEM image of (a) SCU-CPN-1-Re and SCU-CPN-1 after being irradiated by 1000 kGy of (b) β-rays or (c) γ-rays. Scale bar = 1 μm.

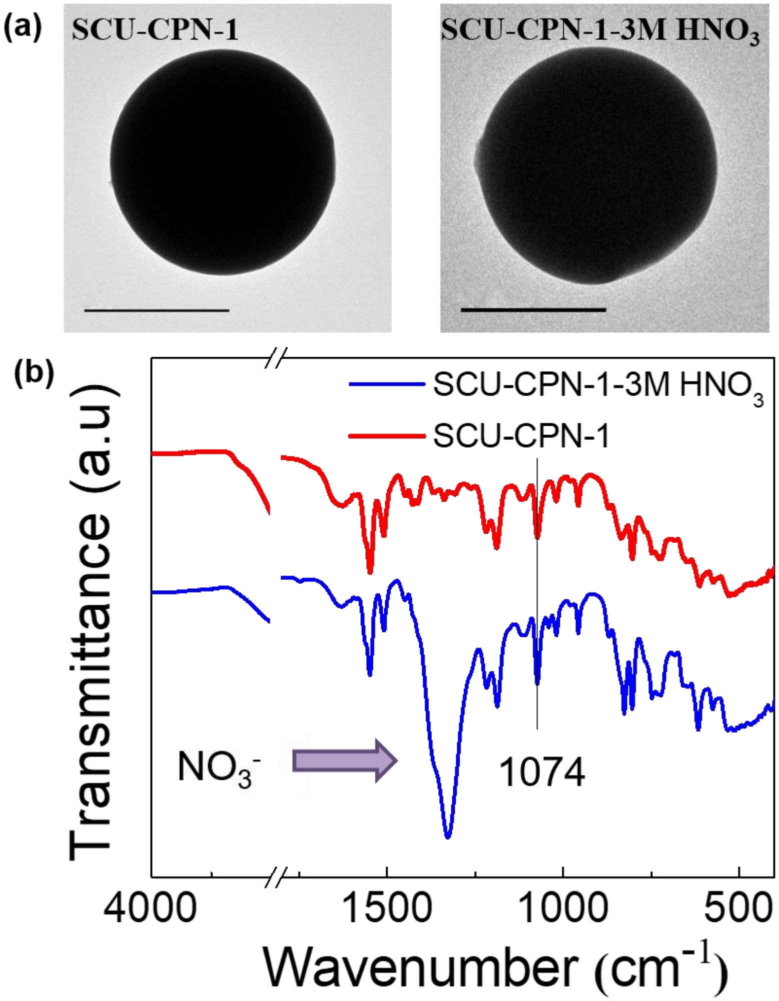

Supplementary Figure 8. TEM image and FT-IR spectra of SCU-CPN-1 and SCU-CPN-3M HNO_3. (a) TEM image and (b) FT-IR spectra of SCU-CPN-1 and SCU-CPN -3M HNO_3 (SCU-CPN-1 materials after being immersed in 3 M HNO_3 solution for 12 h). Scale bar = 1 μm.

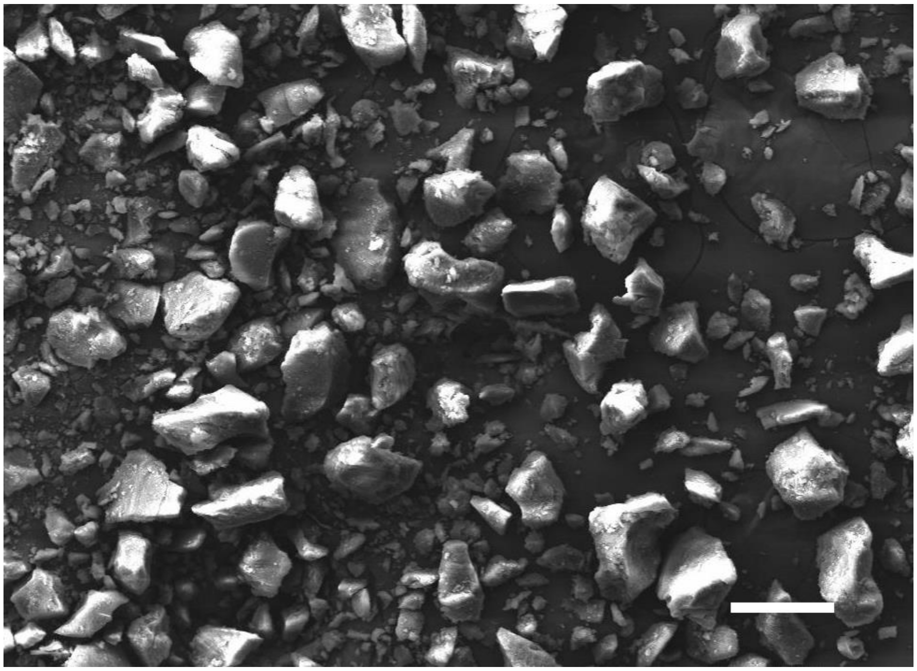

Supplementary Figure 9. SEM image of Purolite A530E-ground. Scale bar = 100 µm.

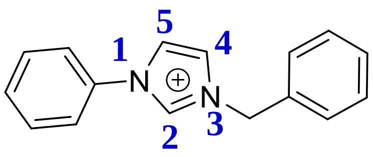

Supplementary Figure 10. The model for describing the local structure of SCU-CPN-1.

Supplementary Table 1. Comparison of equilibrium time and rate constant of SCU-CPN-1 with other materials.

Sorbents	Experimental conditions	Equilibrium time	Rate constant	Ref.
SBN	$[Re]_0$ = 28 mg l^{-1}; pH = 7; 0.5 g l^{-1}; stirring	10 min	--	1
PAF-1-F	Molar ratio ReO_4^-: PAF-1-F = 1:2	250 min	--	2
SCU-100	$[Re]_0$ = 28 mg l^{-1}; pH = 7; 1 g l^{-1}; stirring	30 min	--	4
Acidosasa edulis shoot shell bio-char	T = 298 K; pH = 1; 3 g l^{-1}; $[Re]_0$ = 20 mg l^{-1}	350 min	8.3×10^{-3} g mg^{-1} min^{-1}	5
PS-g-4VP-IE	$[Re]_0$ = 2000 mg l^{-1}; V = 5 ml; $m_{sorbent}$ = 0.1g; T = 298 K	30 min	1.8×10^{-2} min^{-1}	6
PP-g-2-VP	$[Re]_0$ = 320 mg l^{-1}; pH = 2.2	30 min	3.81×10^{-3} s^{-1}	7
ZrO_2@rGO	pH = 4.0 ± 0.1; 0.1mg l^{-1}; $[Re]_0$ = 10.00 mg l^{-1}; T = 293 K	5 h	1.2×10^{-2} g mg^{-1} h^{-1}	8
rGOs	0.1 g l^{-1}; $[Re]_0$ = 20.0 mg l^{-1}; pH = 3.0	10 min	--	9
NZVI	0.1 g l^{-1} $[Re]_0$ = 20.0 mg l^{-1}; pH = 3.0	90 min	3×10^{-3} g mg^{-1} min^{-1}	9
NZVI/rGOs	0.1 g l^{-1}; $[Re]_0$ = 20.0 mg l^{-1}; pH = 3.0	50 min	6.5×10^{-2} g mg^{-1} min^{-1}	9
4-ATR resin	$m_{sorbent}$ = 10.0 mg; $[Re]_0$ = 7.2 mg/50.0 ml; pH = 2.6; 100 rpm	8 h	8.2×10^{-5} s^{-1}	10
R_2SO_4 resin	$[Re]_0$ = 200 mg l^{-1}, V = 15 ml; $m_{sorbent}$ = 0.02 g; pH = 6.25; room temperature	60 min	1.65×10^{-2} min^{-1}	11
D318 Resin	$m_{sorbent}$ = 50.0 mg; $[Re]_0$ = 320 mg l^{-1}; T = 298 K; V = 50.0 ml	115 min	6.37×10^{-4} s^{-1}	12
Purolite A532E	$[Re]_0$ = 28 mg l^{-1}; pH = 7; 1 g l^{-1}; stirring	150 min	4.60×10^{-3} g $mg^{-1}min^{-1}$	13
Purolite A530E	$[Re]_0$ = 28 mg l^{-1}; pH = 7; 1 g l^{-1}; stirring	150 min	6.75×10^{-3} g $mg^{-1}min^{-1}$	13
UiO-66-NH_3^+	molar ratio ReO_4^-/ UiO-66-NH_3^+ = 1:2	>24 h	--	14
SCU-101	$[Re]_0$ = 28 mg l^{-1}; pH = 7; 1 g l^{-1}; stirring	10 min	--	15
SCU-CPN-1	$[Re]_0$ = 28 mg l^{-1}; pH = 7; 1 g l^{-1}; stirring	30 s	--	This work
Purolite A530E-ground	$[Re]_0$ = 14 mg l^{-1}; pH = 7; 0.05 g l^{-1}; stirring	140 min	6.46×10^{-4} g mg^{-1} min^{-1}	This work
SCU-CPN-1	$[Re]_0$ = 14 mg l^{-1}; pH = 7; 0.05 g l^{-1}; stirring	10 min	2.01×10^{-2} g mg^{-1} min^{-1}	This work

Supplementary Table 2. Fitting results based on the Pseudo-first-order kinetic and Pseudo-second-order kinetic models.

Material	Pseudo-first-order kinetic model			Pseudo-second-order kinetic model		
	q_e (mg g^{-1})	k_1 (min^{-1})	R^2	q_e (mg g^{-1})	k_2 (g g^{-1} min^{-1})	R^2
SCU-CPN-1	23.79	9×10^{-3}	0.78	384.61	2.01×10^{-2}	>0.99
A530E-ground	112.04	1.37×10^{-2}	0.87	367.65	6.46×10^{-4}	>0.99

Supplementary Table 3. Comparison of the distribution coefficient (K_d) by cationic materials.

Cationic materials	K_d (ml g^{-1})	Ref.
Mg-Al-LDH	262	1
NDTB-1	652	1
$Y_2(OH)_5Cl$	112	1
$Yb_3O(OH)_6Cl$	120	1
PAF-1-F	2.55×10^4	2
SCU-6	3.0×10^3	3
SCU-7	63	3
SCU-100	3.3×10^5	4
**SCU-CPN-1**	**6.2×10^5**	**This work**

Supplementary Table 4. Fitting results based on the Langmuir and Freundlich models.

Sample	Langmuir			Freundlich		
	q_m (mg g^{-1})	K_L (l mg^{-1})	R^2	k_F (L^n mol^{1-n} g)	n	R^2
SCU-CPN-1	876	0.134	>0.99	554013	1.69	0.79

Supplementary Table 5. Sorption isotherm of SCU-CPN-1 with excess of ReO_4^- .

Material	C_0(Re) (ppm)	Sorption Capacity (mg ReO_4^-/g sorbent)
SCU-CPN-1	2000	948±20
	4000	999±20

Supplementary Table 6. Comparison of the maximum ReO_4^- sorption capacities of SCU-CPN-1 with other cationic materials.

Category	Sorbents	Experimental conditions	Capacity (mg g^{-1})	Ref.
Inorganic materials	Nano SiO_2	T=298 K; pH=2	4.94	16
	Biochar	T=298 K; 12 h; 3 g l^{-1}	46.5	7
	$Yb_3O(OH)_6Cl$	Ambient temperature; pH=7.0±0.1; 0.5 g l^{-1}	48.6	1
	NDTB-1	Ambient temperature; pH=7.0±0.1; 0.5 g l^{-1}	49.4	1
	LDHs	Ambient temperature; pH=7.0±0.1; 0.5 g l^{-1}	**130.2**	1
Composites	ZrO_2@rGO	T=303 K; 24 h	43.55	8
	NZVI/rGOs	0.1 g l^{-1}; pH=5	85.77	9
	GO-DEA-DIBA	303 K; 2 g l^{-1}; 48 h	**140.82**	17
Resins	PP-g-2-VP	pH=2.2; appropriate temperature	113	7
	PS-g-4VP-IE	Ambient temperature; 2 h	252	6
	D318 resin	T=298 K; pH=5.2; 1 g l^{-1}	351	12
	4-ATR resin	T=298 K; 8 h; pH 2.6; 10 h	354	10
	R_2SO_4 resin	Ambient temperature; 1.3 g l^{-1}; pH=6.25; 4 h	462	11
	Purolite A532E	Ambient temperature; pH=7.0±0.1; 0.5 g l^{-1}	446	13
	Purolite A530E	Ambient temperature; pH=7.0±0.1; 0.5 g l^{-1}	**706**	13
MOFs	UiO-66-NH_3^+	24 h	159	14
	SCU-101	Ambient temperature; pH=7.0±0.1; 1 g l^{-1}	217	15
	SCU-100	Ambient temperature; pH=7.0±0.1; 1 g l^{-1}	541	4
	SLUG-21	Ambient temperature; 48 h; 1.6 g l^{-1}	602	18
	SBN	Ambient temperature; pH=7.0±0.1; 0.5 g l^{-1}	**786**	1
CPNs	PAF-1-F	Molar ratio ReO_4^-: PAF-1-F =1:2; 24 h	420	2
	SCU-CPN-1	Ambient temperature; pH=7.0±0.1; 1 g l^{-1}	**999**	This work

Supplementary Table 7. Composition of Hanford Low Activity Waste (LAW) Melter Recycle Stream.

Anions	Concentration (mol l^{-1})	Anion:TcO_4^- molar ratio
TcO_4^-	1.94×10^{-4}	1.0
NO_3^-	6.07×10^{-2}	314
Cl^-	6.39×10^{-2}	330
NO_2^-	1.69×10^{-1}	873
SO_4^{2-}	6.64×10^{-6}	0.0343
CO_3^{2-}	4.30×10^{-5}	0.222

Supplementary Table 8. Results of ReO_4^- sorption by SCU-CPN-1 from simulated Hanford waste.

Simulated wastes	Anions	Solid-to-liquid ratio (g l^{-1})	Anion removal percentage (%)
Hanford waste	ReO_4^-	1:1	52.0±5
	ReO_4^-	5:1	83.7±5
	TcO_4^-	5:1	89.5±5

Supplementary Table 9. Results of ReO_4^- desorption from sorbed SCU-CPN-1 at different temperature.

Desorption temperature	Concentration of desorption solutions of NaCl (mol l^{-1})	Desorption percentage (%)
25 °C	1	64.59
	1.5	71.86
	2	74.49
60 °C	0.1	46.48
	1	87.82
	2	93.68
	4	94.90
80 °C	0.1	53.61
	1	97.85
	2	98.07
	4	98.08

Supplementary Table 10. EXAFS results of Re (VII) adsorption by SCU-CPN-1at Re L_{III}-edge, T=298 K. C.N. means coordination number of the neighbors, R is the bond distance, and σ^2 is the Debye-Waller factor.

Sample	shell	C.N.	R(Å)	σ^2 (Å^2)
SCU-CPN-1-Re	Re=O	4.1	1.73	0.024

Supplementary References:

1. Zhu, L., Xiao, C., Dai, X., Li, J., Gui, D., Sheng, D., Chen, L., Zhou, R., Chai, Z., Albrecht-Schmitt, T. E., Wang, S. Exceptional perrhenate/pertechnetate uptake and subsequent immobilization by a low-dimensional cationic coordination polymer: overcoming the hofmeister bias selectivity. *Environ. Sci. Technol. Lett.* **4**, 316-322 (2017).
2. Banerjee, D., Elsaidi, S. K., Aguila, B., Li, B., Kim, D., Schweiger, M. J., Kruger, A. A., Doonan, C. J., Ma, S., Thallapally, P. K. Removal of pertechnetate-related oxyanions from solution using functionalized hierarchical porous frameworks. *Chem. Eur. J.* **22**, 17581-17584 (2016).
3. Bai, Z., Wang, Y., Li, Y., Liu, W., Chen, L., Sheng, D., Diwu, J., Chai, Z., Albrecht-Schmitt, T. E., Wang, S. First cationic uranyl–organic framework with anion-exchange capabilities. *Inorg. Chem.* **55**, 6358-6360 (2016).
4. Sheng, D., Zhu, L., Xu, C., Xiao, C., Wang, Y., Wang, Y., Chen, L., Diwu, J., Chen, J., Chai, Z., Albrecht-Schmitt, T. E., Wang, S. Efficient and selective uptake of TcO_4^- by a cationic metal–organic framework material with open Ag^+ sites. *Environ. Sci. Technol.* **51**, 3471-3479 (2017).
5. Hu, H., Jiang, B., Zhang, J., Chen, X. Adsorption of perrhenate ion by bio-char produced from acidosasa edulis shoot shell in aqueous solution. *RSC. Adv.* **5**, 104769-104778 (2015).
6. Zu, J., Ye, M., Wang, P., Tang, F., He, L. Design of a strong-base anion exchanger and its adsorption and elution behavior for rhenium(VII). *RSC Adv.* **6**, 18868-18873 (2016).
7. Zu, J. H., Wei, Y. Z., Ye, M. S., Tang, F. D., He, L. F., Liu, R. Q. Preparation of a new anion exchanger by pre-irradiation grafting technique and its adsorptive removal of rhenium(VII) as analogue to ^{99}Tc. *Nucl. Sci. Tech.* **26**, 69-75 (2015).
8. Gao, Y., Chen, K., Tan, X., Wang, X., Alsaedi, A., Hayat, T., Chen, C. Interaction mechanism of Re(VII) with zirconium dioxide nanoparticles archored onto reduced graphene oxides. *ACS Sustainable Chem. Eng.* **5**, 2163-2171 (2017).

9. Li, J., Chen, C., Zhang, R., Wang, X. Reductive immobilization of Re(VII) by graphene modified nanoscale zero-valent iron particles using a plasma technique. *Sci. China Chem.* **59**, 150-158 (2016).
10. Xiong, C., Yao, C., Wu, X. Adsorption of rhenium(VII) on 4-amino-1,2,4-triazole resin. *Hydrometallurgy* **90**, 221-226 (2008).
11. Jia, M., Cui, H., Jin, W., Zhu, L., Liu, Y., Chen, J. Adsorption and separation of rhenium(VII) using N-methylimidazolium functionalized strong basic anion exchange resin. *J. Chem. Technol. Biot.* **88**, 437-443 (2013).
12. Shu, Z., Yang, M. Adsorption of rhenium(VII) with anion exchange resin D318. *Chinese. J. Chem. Eng.* **18**, 372-376 (2010).
13. Li, J., Zhu, L., Xiao, C., Chen, L., Chai, Z., Wang, S. Efficient uptake of perrhenate/pertechnenate from aqueous solutions by the bifunctional anion-exchange resin. *Radiochim. Acta* DOI: 10.1515/ract-2017-2829.
14. Banerjee, D., Xu, W., Nie, Z., Johnson, L. E. V., Coghlan, C., Sushko, M. L., Kim, D., Schweiger, M. J., Kruger, A. A., Doonan, C. J., Thallapally, P. K. Zirconium-based metal–organic framework for removal of perrhenate from water. *Inorg. Chem.* **55**, 8241-8243 (2016).
15. Zhu, L., Sheng, D., Xu, C., Dai, X., Silver, M. A., Li, J., Li, P., Wang, D., Wang, Y., Chen, L., Xiao, C., Chen, J., Zhou, R., Zhang, C., Farha, O. K., Chai, Z., Albrecht-Schmitt, T. E., Wang, S. Identifying the recognition site for selective trapping of $^{99}TcO_4^-$ in a hydrolytically stable and radiation resistant cationic metal–organic framework. *J. Am. Chem. Soc.* **139**, 14873-14876 (2017).
16. Li, Y., Wang, Q., Li, Q., Zhang, Z., Zhang, L., Liu, X. Simultaneous speciation of inorganic rhenium and molybdenum in the industrial wastewater by amino-functionalized nano-SiO_2. *J. Taiwan. Inst. Chem. E.* **55**, 126-132 (2015).
17. Cui, X., Zhang, P., Wang, Y., Lou, Z., Shan, W. Improving Re(VII) adsorption on diisobutylamine-functionalized graphene oxide. *ACS Sustainable Chem. Eng.* **5**, 1010-1018 (2017).
18. Fei, H., Rogow, D. L., Oliver, S. R. Reversible anion exchange and catalytic properties of two cationic metal−organic frameworks based on Cu(I) and Ag(I). *J. Am. Chem. Soc.* **132**, 7202-7209 (2010).
